# Recombinant expression of single eukaryotic-like proteins changes bacteria-sponge interactions

**DOI:** 10.1093/ismeco/ycag304

**Published:** 2026-10-22

**Authors:** Yibo Wu, Yunke Jia, Joachim S Larsen, Torsten Thomas

**Affiliations:** Centre for Marine Science and Innovation & School of Biological, Earth and Environmental Sciences, The University of New South Wales, Sydney, New South Wales 2052, Australia; Centre for Marine Science and Innovation & School of Biological, Earth and Environmental Sciences, The University of New South Wales, Sydney, New South Wales 2052, Australia; School of Environmental and Life Sciences, University of Newcastle, Newcastle, New South Wales 2308, Australia; Centre for Marine Science and Innovation & School of Biological, Earth and Environmental Sciences, The University of New South Wales, Sydney, New South Wales 2052, Australia

**Keywords:** bacteria-host interactions, *Synechococcus,* ankyrin repeat, tetratricopeptide repeat, NHL domain, choanocyte

## Abstract

Eukaryotic-like proteins (ELP) are often found in intracellular symbionts of insects, plants, and evolutionary early branching sponges, however their functions remain largely uncharacterized. Here we show that the recombinant expression of single genes of various ELP classes can confer on free-living cyanobacteria the capacity to escape phagocytosis by sponges and thus enable intracellular survival. The findings establish ELPs as molecular factors that can control sponge-microbe interactions, which has implications for the evolution of microbial symbiosis.

Symbiotic interactions with microorganisms are essential for the function, adaptation, and evolution of metazoans [1, 2]. As early branching metazoan, sponges (phylum Porifera) are important models for the comparative study of host–microorganism interactions and the evolution of mechanisms controlling symbiosis [3, 4]. Mechanisms to control symbiotic interactions are particularly important for sponges, because they satisfy a significant amount of their nutritional requirements by pumping large volumes of the surrounding seawater through their bodies in order to filter, phagocytose, and digest diverse microorganisms as food [5, 6]. Yet, sponges maintain stable, complex and often highly specific communities of symbionts, which have essential functions for their health and performance, in their bodies, including in the extracellular mesohyl matrix or within cells [7–9]. Sponges can discriminate between symbiont consortia and food bacteria when supplied in the surrounding water, because the former have been found to be consumed to a lesser extent by the sponge *Aplysina aerophoba* [10]. This discrimination has been recently correlated with the differential expression of nucleotide oligomerization domain (NOD)-like receptors (NLRs) that are part of the innate immune system [11]. However, the precise mechanisms underlying this discrimination remains poorly understood, and potentially involves other host-controlled processes (eg, production of antimicrobial peptides) or symbiont-derived factors that influence phagocytosis [12–15].

Metagenomic analyses have revealed the unexpected abundance and prevalence of genes encoding eukaryotic-like protein (ELP) in taxonomically diverse sponge symbionts [14, 16–18]. ELPs are characterized by protein domains, such as ankyrin repeats (ANK) or tetratricopeptide repeats (TPR), that are typically found in eukaryotes and are often involved in protein–protein interactions [19, 20]. Some ELPs from uncultured sponge symbionts, when recombinantly expressed in *Escherichia coli*, have been found to lead to the accumulation and persistence of bacteria inside the free-living amoeba *Acanthamoeba castellanii*, indicating a role in modulating eukaryotic phagocytosis [20, 21]. Similarly, an ANK-type ELP from a bacteriophage found in a sponge protected *E. coli* against human macrophage phagocytosis [22]. The results indicate that ELP might be a key factor that allows symbionts to modulate the phagocytosis by sponges, however direct experimental evidence for this is currently lacking.

Here we provide direct experimental proof for the role of ELPs in sponge-microbe symbiosis by recombinantly expressing them in *Synechococcus elongatus* PCC 7942 and investigate interactions with a sponge. The free-living, freshwater *S. elongatus* PCC 7942 is readily genetically tractable, including with expression plasmids [23], and has similar morphological properties to *Synechococcus* strains that are abundant in the marine environment [24–27]. *Synechococcus* cells are thus an ecologically relevant food sources for sponges [28], yet some lineages (eg, *S. spongarium*) have evolved to become sponge symbionts that also encode ELPs [29]. We focused on different ELP classes found in bacterial symbionts of the sponge species *Cymbastela concentrica* and *Cymbastela coralliophila* (Fig. 1a) [20, 21]. These include two different ANK proteins (ELP1 and SSA4), a TPR protein (ELP5) and a NHL-domain protein (ELP9), whose recombinant expressions in *E. coli* have been previously found to lead to intracellular, bacterial accumulation in amoeba [20, 21]. A fifth protein with cadherin and phenylalanyl-glycyl and glycyl-alanyl-prolyl repeat domains (CAD42) was also analyzed as it was one of the most highly expressed ELPs in a metatranscriptomic analysis of the microbiome of *C. concentrica* [30].

**Figure 1:**
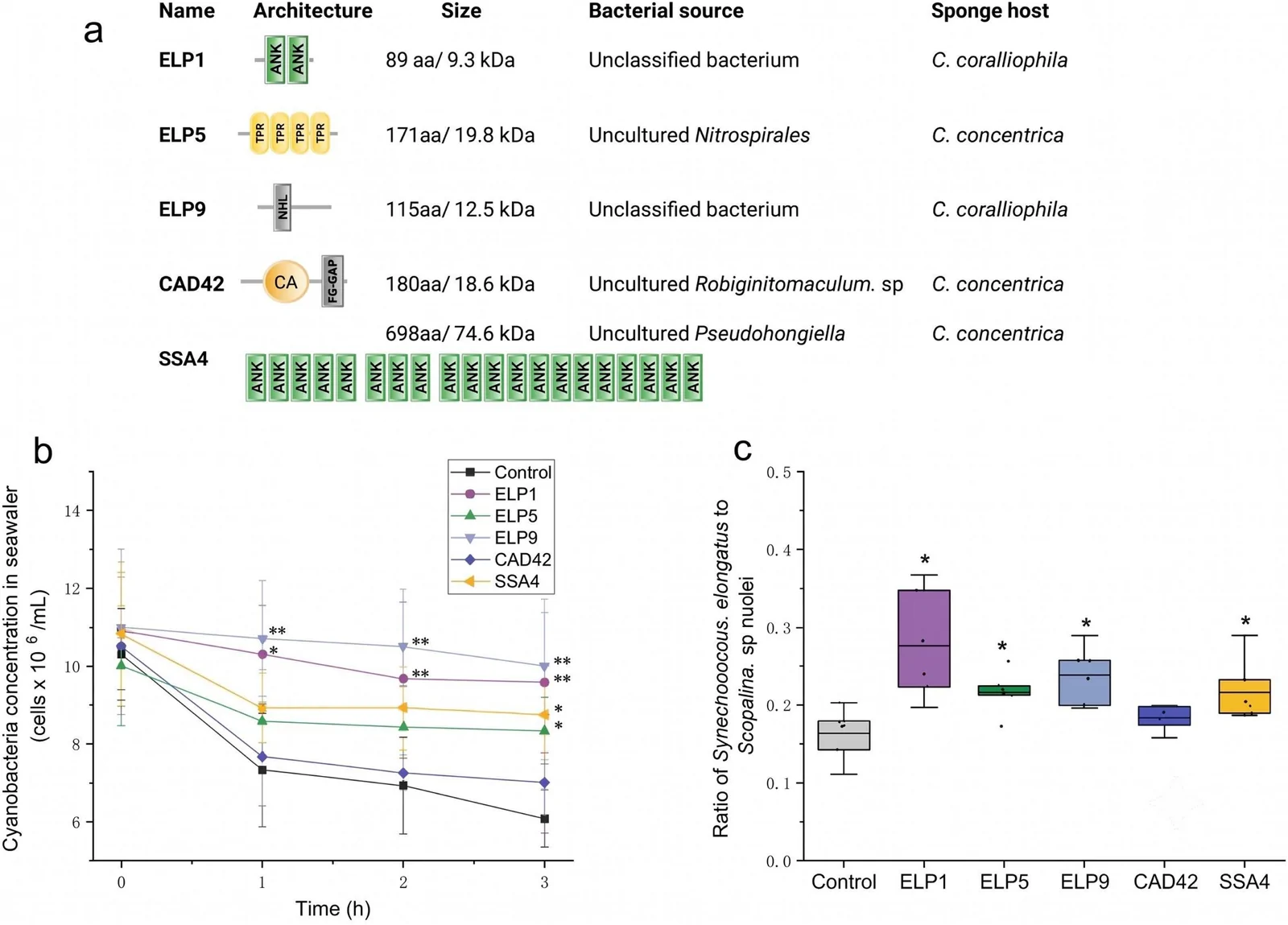
(a) ELPs used in this study. Domain architecture refers to ankyrin repeats (ANK), tetratricopeptide repeats (TPR), NCL-1, HT2A and Lin-41 repeats (NHL), cadherin repeats (CA), and the phenylalanyl-glycyl and glycyl-alanyl-prolyl (FG-GAP) repeats. Domain structures were defined with the SMART database [51]; (b) The concentration of *S. elongatus* expressing either no recombinant protein, ELP1, ELP5, ELP9, CAD42, or SSA4 in seawater when incubated with explants of *Scopalina* sp. The data are from two independent experiments each with three biological replicates. Error bars represent the standard error of the mean. Pairwise contrasts were assessed by Poisson GLM followed by Dunnett’s post-hoc tests, comparing each ELP-expressing strain against the control (i.e. *S. elongatus* with empty pSyn_6). An image of a cell count is shown in Figure S5; (c) The ratio between the number of *S. elongatus* and *Scopalina* sp. nuclei in sponge tissue sections for *S. elongatus* expressing either no recombinant protein (grey), ELP1 (purple), ELP5 (green), ELP9 (light blue), CAD42 (dark blue), or SSA4 (yellow). Data are from timepoint 3 h for two independent experiments, each performed in triplicate. Pairwise contrasts against the negative control were evaluated by Poisson generalized linear model (GLM) with Wald χ^2^ tests. *P*-value ≤.05, *P*-value ≤.005.

The genes for the five ELPs were cloned into the pSyn_6 expression vector under the control of the strong constitutive *psbA* promoter and transformed into *S. elongatus* PCC 7942 (see Materials and Methods in Supplementary Information). Reverse transcription polymerase chain reaction showed full-length expression of the genes (Supplementary Information, Fig. S1). These recombinant strains, including one with an empty pSyn_6 vector as control, were then transferred to seawater containing explants of the low-microbial abundance (LMA) sponge *Scopalina* sp., which has no detectable cyanobacterial symbionts based on microscopy (Fig. S2) and previous metagenomic sequencing [16]. *S. elongatus* was found to be stable in seawater and only decreased in concentration when *Scopalina* sp. explants were present, showing active bacterial removal by the sponge (Fig. S3). Furthermore, a removal rate of 1.39 ± 0.29 × 10^6^ cells·mL^−1^·h^−1^ was observed for *S. elongatus* pSyn_6 (control) over 3 h (Fig. 1b), which is estimated to result in 1.2 × 10^9^ cyanobacterial cells being removed per hour per gram sponge dry weight (see Material and Methods). Comparable measurements on removal rates for other LMA sponges are rare, with only one study on *Dysidea avara* reporting a removal rate of 7 × 10^8^ bacteria per gram dry weight per hour [31], similar to our rate.

At 3 h the average removal rates of strains expressing ELP1, ELP5, ELP9, and SSA4 were 0.44 ± 0.33 × 10^6^, 0.56 ± 0.36 × 10^6^, 0.33 ± 0.41 × 10^6^ and 0.69 ± 0.29 × 10^6^ cells·mL^−1^·h^−1^, respectively, which are significantly lower than for *S. elongatus* pSyn_6 (control) (time-stratified Dunnett’s comparisons). This also resulted in significantly higher residual cell concentrations at 3 h for strains expressing ELP1, ELP5, ELP9, and SSA4 (Fig. 1b). In contrast, the removal rate and residual cell concentration at 3 h was not different for the strain expressing CAD42 and the control.

A reduced removal rate of *S. elongatus* from seawater could result from two distinct mechanisms: (a) a reduction in sponge’s pumping activity, leading to reduced uptake of bacterial cells into the sponge’s interior and hence continuing high concentrations in seawater; or (b) normal pumping activity with impaired phagocytosis or digestion of *S. elongatus* cells, allowing them to survive in the sponge tissue and continuously being expelled again into the seawater by the water pumping. To distinguish between these two possibilities, quantitative microscopy was used to determine the *S. elongatus* concentrations within the sponge tissue (see Materials and Methods for details).

*Scopalina* sp. exposed to *S. elongatus* expressing ELP1, ELP5, ELP9, and SSA4 showed a significantly higher ratio of internal cyanobacteria to sponge nuclei compared the control group (Fig. 1c). A higher internal *S. elongatus* concentration relative to the control is not consistent with the first mechanism postulated above, but instead supports the second, that is, cyanobacteria are being still taken up by the sponge, but survive in the interior allowing them to leave the sponge again with the water flow. No significant difference in the ratio was found for *S. elongatus* expressing CAD42 compared to the control, consistent with the strain also showing no impact on the removal rate from seawater (Fig. 1b).

To understand if this survival is due to an impact on phagocytosis, we quantify the localization of cyanobacteria within the sponge tissue using confocal microscopy and Z-stack analyses (see Material and Methods). This showed that the vast majority of cyanobacterial cells were intracellular (Fig. 2) and we could not detect any significant differences between cyanobacteria containing recombinant ELPs or the empty pSyn_6 vector (control). This indicates that the accumulation of cyanobacteria across the sponge tissue (Fig. 1c) involves a normal bacterial uptake via phagocytosis (i.e. bacteria do not accumulate extracellularly), but that overall more sponge cells contain intracellular cyanobacteria when those recombinantly express ELP1, ELP5, ELP9, or SSA4 (Fig. 1c). This is consistent with previous observations that the expression of ELP1, ELP5, ELP9, or SSA4 in *E. coli* causes more amoeba within a population to contain intracellular bacteria [20, 21]. As expression of these ELPs also decreased the removal rate from seawater (Fig. 1b), these results further suggest an impairment of sponge cells to complete the phagocytosis process in our feeding assays.

**Figure 2:**
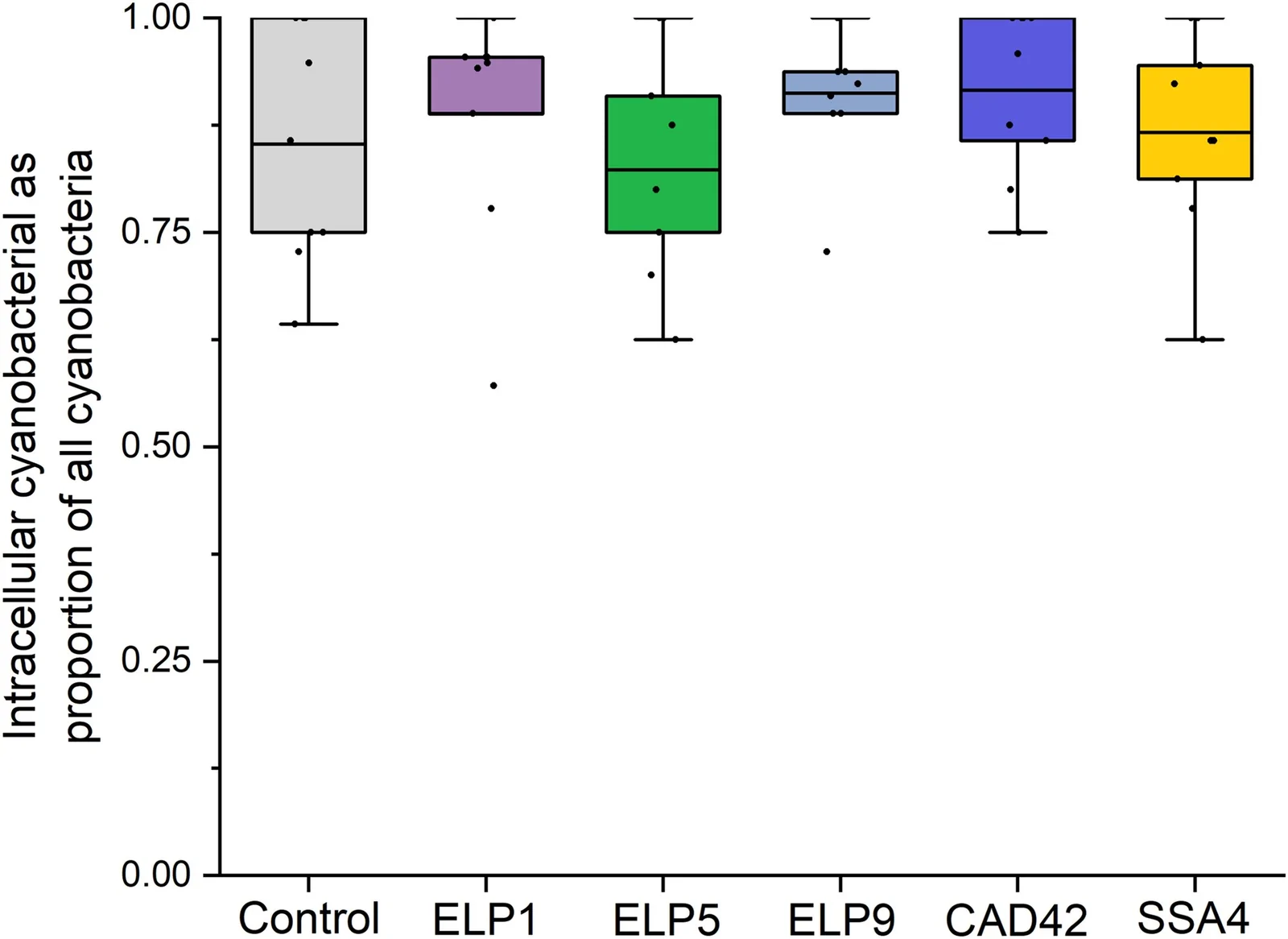
Proportion of intracellular cyanobacteria without (control) or with recombinant ELPs in the tissue of *Scopalina* sp. after 3 h exposure (see Fig. 1b). Localization of an average of 15.4 (range 4–30) cyanobacterial cells per image (n = 9) were determined. Technical variability between cryosections was assessed using coefficient of variation, which were below 26% for every given biological replicate. No significant differences were detected between cyanobacteria containing recombinant ELPs or the pSyn_6 control (one-way ANOVA, *F* (5,48) = 0.823, *P*-value = .540). Variance homogeneity was confirmed by Levene’s test (*P*-value = .311). Representative images can be found in Fig. S5 and Supplementary Movies S1 and S2.

Qualitative microscopy also showed that strains expressing ELP1, ELP5, ELP9, and SSA4 tended to be localized around choanocyte chambers (dashed lines in Figs 3 and S4), whereas the control and CAD42-expressing *S. elongatus* cells appear to be more diffusely distributed across the sponge tissue. Choanocyte chambers are the principal feeding sites of the sponge’s aquiferous system, where microbial particles that arrive via incurrent canals are captured by choanocytes [32, 33]. Captured particles are then transferred via transcytosis to the mesohyl where they are ultimately phagocytosed and digested by archaeocytes [10, 34, 35]. Intracellular cyanobacteria expressing ELP1, ELP5, ELP9, and SSA4 may thus disrupt the transcytosis process in choanocytes, however further work is required to fully understand how ELP expression impact the various phagocytic cell types in sponges.

**Figure 3:**
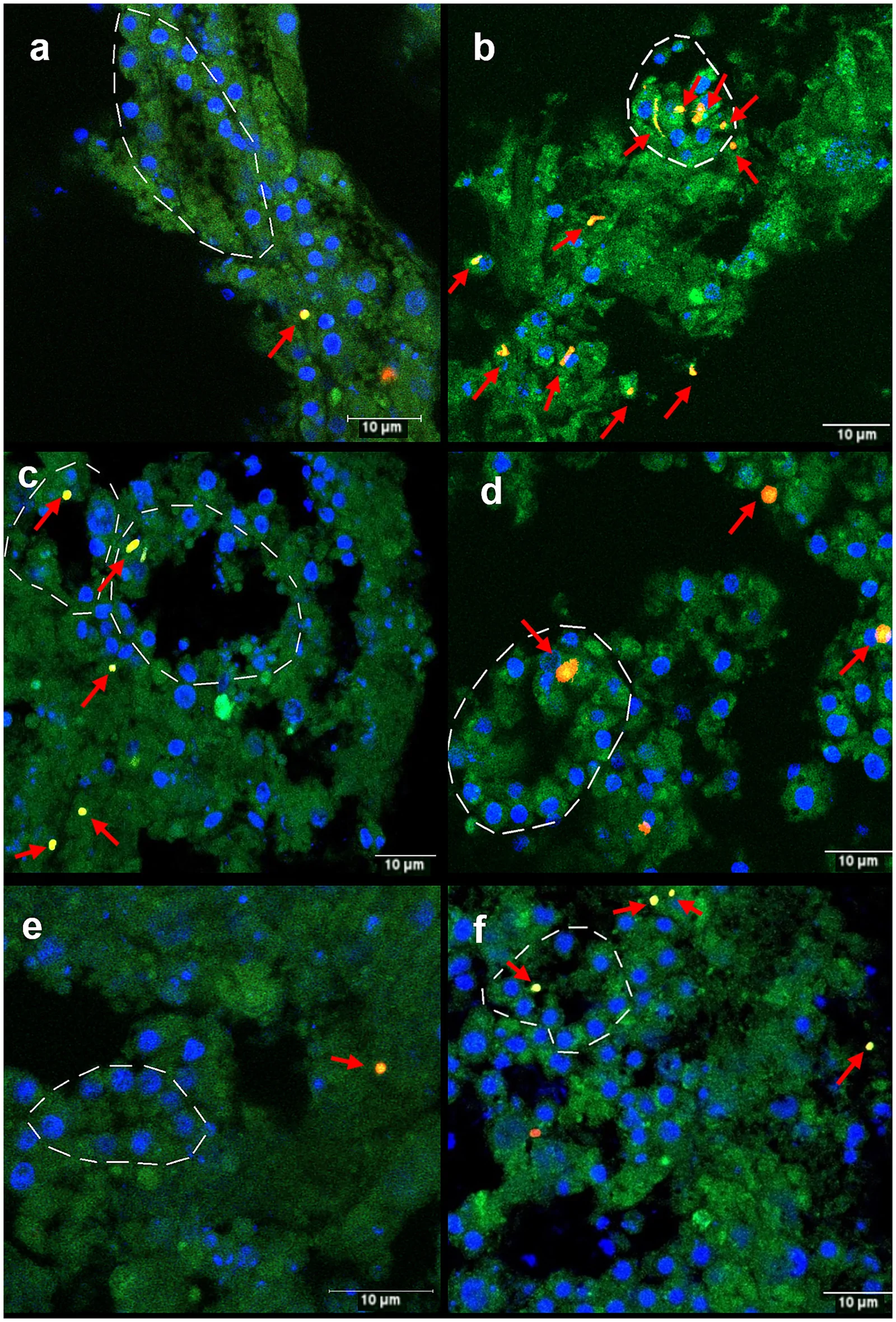
Representative images showing the localization of *S. elongatus* expressing either no recombinant protein (a), ELP1 (b), ELP5 (c), ELP9 (d), CAD42 (e), or SSA4 (f), within *Scopalina* sp. tissue. Red arrows indicate cyanobacteria. Due to the natural fluorescence of cyanobacteria appeared yellow to orange, and variations in size is due to image focus. Dashed lines outline choanocyte chambers with sponge nuclei (blue) stained with 4′,6-diamidino-2-phenylindole. Images were taken 3 h after adding bacteria to sponges. Scale bar 10 μm. More images can be found in the Supplementary Information Fig. S3.

ELPs are not only common to sponge symbionts, but their genes are also found in intracellular bacterial symbionts of amoeba [36] and fungi [37], intracellular bacterial parasites of insects [38, 39], plant-associated rhizobia [40] and the plant-nucleus associated *Methylobacterium extorquens* [41, 42]. Whilst a common theme in all these systems is the intracellular localisation of bacteria, which in some cases also includes phagocytic processes (e.g. in amoeba), none of the ELPs in these systems have been functionally characterized. However, ELPs have been studied in intracellular pathogens of humans. For example, AnkX, an ankyrin repeat-containing effector protein from *Legionella pneumophila*, catalyzes the phosphocholination of host Rab GTPases thereby arresting phagolysosomal maturation and facilitating bacterial survival [43–45]. The TPR-containing serine/threonine kinase PknG from *Mycobacterium tuberculosis* binds host RAB14 and inhibits phagosome–lysosome fusion, thereby blocking phagosomes maturation [46, 47]. And finally, the *M. tuberculosis* protein PknD from Ser/Thr protein kinase (STPK) family, which contains an NHL domain, adheres to extracellular matrix proteins of the host thereby promoting bacterial survival [48–50]. Together with our results this demonstrates the conserved function of some ELP classes in controlling phagocytosis processes and intracellular bacterial persistence in hosts across a wide spectrum of evolutionary divergence (e.g. from sponges to humans).

Our results also demonstrate that the introduction of a single ELP gene can change the interactions of a free-living bacterium with a sponge host. Phylogenetic analysis has provided support that ELPs from sponge symbionts have been acquired from their host and/or have undergone secondary symbiont-symbiont transfers [20]. ELPs have also been identified on mobile genetic elements involved in horizontal genes transfer [22] and this could facilitate a rapid adaptation of a non-symbiont to overcome the phagocytosis pressure of the sponge host and allow for extended persistence in the tissue. Whether this process can also aid with the adaptation across host systems remains to be explored, however the fact that some of the ELPs studied here (ELP1, ELP5, ELP9, and SSA4) also impact amoebal phagocytosis [20, 21], would support this possibility.

Full and stable integration of a free-living bacterium into the sponge holobiont would also require adaptations that allow, for example, for metabolic interactions with the host and other microbiome members as well as defensive response to the innate immune system. The order in which these adaptions occur need to be further explored, however our results show that ELPs are one important factor that supports the evolution of symbionts in these early branching metazoan group of sponges.

## Supplementary Material

Supplementary_files_ycag304

## Data Availability

All data generated or analyzed during this study are included in this published article and its supplementary information files.
