# Supplementary material for: Recombinant expression of single eukaryotic-like proteins changes bacteria-sponge interactions
Source: ISME Commun. 2026 Oct 22;6(1):ycag304. doi: 10.1093/ismeco/ycag304 (PMC13747020; doi:10.1093/ismeco/ycag304)
Supplement: Supplementary file 1 [file supplementary_files_ycag304.zip › Wu_etal_Supplementary_Information_revised_ISME_Comm_final.docx]

**Materials and Methods**

**Organisms and cultivation**

The *Synechococcus* *elongatus* PCC 7942 was kindly provided by Professor Brett Neilan from the University of Newcastle, School of Environmental and Life Sciences, Australia. The 50× cyanobacteria BG-11 media (Sigma-Aldrich, St. Louis, MO, USA) was used to cultivate *S. elongatus*. The cultures were maintained at 30°C under a cycle of 12 hours with 50 μmol photons m^-2^ s^-1^ and 12 hours of darkness with gentle shaking at 100 rpm.

**Construction of expression plasmids**

The gene *ssa4* for SSA4 was obtained from a previously described pBAD plasmid construct [1]. The gene sequences for the other four ELPs were chemically synthesized and cloned into the pET-28a (+) plasmid by the company Agentide Inc. (New Jersey, USA). The recombinant plasmids served as templates for PCR amplification to subclone the genes with primer pairs designed to incorporate appropriate restriction sites (*Eco*RI, *Nde*I and *Bgl*II) (Table 1).

**Table 1:** Information of primers and restriction sites (underlined and names in brackets) used in the present study:

| Name | Sequence |
| --- | --- |
|  |  |
| T7 | TAATACGACTCACTATAGGG |
| T7t | GCTAGTTATTGCTCAGCGG |
| pSyn_6-F | TCTCCTAGCCTAAAAAGCAAG |
| pSyn_6-F2 | GAGGCATTTCTGTCCTGGCT |
| pSyn_6-R | GTCGAAAGACTGGGCCTTCT |
| pSyn_6-R2 | AGAAGGCCCAGTCTTTCGAC |
|  |  |
| ELP1- F | GGAATTCCATATGATGCTGCTGTCTGAGTTTGGAG (*Nde* I) |
| ELP1- R | CCGGAATTCTTAGTTGTGTGTGCACCCACC (*Eco*R I) |
| ELP5- F | GAAGATCTATGGATTTGCAAGATTTTGTCCAAC (*Bgl* II) |
| ELP5- R | CCGGAATTCTCAATTCATTAAACCAATAAGCCGCATC (*Eco*R I) |
| ELP9- F | GGAATTCCATATGCAAGTTTTCAATGCTGATGG (*Nde* I) |
| ELP9- R | CCGGAATTCTTAGCACAATACAAATAGTACATAGCAATAC (*Eco*R I) |
| SSA4- F | GGAATTCCATATGGATGTGAAGAGTAATAATGACTGG (*Nde* I) |
| SSA4- R | CCGGAATTCTCATTGATTGTTCTGCACATCCAG (*Eco*R I) |
| Cad42- F | GGAATTCCATATGATGCGCATTGAGGATGGGGTG (*Nde* I) |
| Cad42- R | CCGGAATTCCTAGGCCCCGCCATAGATG (*Eco*R I) |

PCRs were set up in a total volume of 20 μL containing: 10 ng of plasmid DNA, 10 μM of each forward and reverse primer, 10 μl of 2 × PCR Master Mix (Thermo Fisher Scientific, Waltham, MA, USA). The cycling conditions were set as follows: initial denaturation at 94°C for 2 mins, followed by 30 cycles of denaturation at 98°C for 30 seconds, annealing at 55°C for 30 seconds, and extension at 72°C for 1 minute, with a final extension step at 72°C for 5 minutes. Due to the repetitive nature of the *ssa4* gene, a modified amplification protocol was applied: initial denaturation at 94°C for 2 minutes; five cycles of 30 seconds at 94°C, 20 seconds at 70°C, and 3 minutes at 72°C; followed by 25 cycles of 30 seconds at 94°C, 20 seconds at 50°C, and 3 minutes at 72°C; with a final extension at 72°C for 5 minutes.

The PCR products were evaluated by 1.2% (w/v) agarose gel electrophoresis and purified using a QIAquick PCR Purification Kit (Qiagen, Hilden, Germany). Due to some unspecific amplification, the appropriate PCR band for the *ssa4* gene was isolated with the QIAquick Gel Extraction Kit (Qiagen). The purified amplicons were digested using either *Eco*RI, *Nde*I, or *Bgl*II according to manufacturer’s instructions (Thermo Fisher Scientific), purified as above and subsequently ligated into the GeneArt™ *Synechococcus* Protein Expression Vector pSyn_6 (Thermo Fisher Scientific), which had been linearized with corresponding restriction enzymes. The ligation was performed using the Anza™ T4 DNA Ligase Master Mix following the manufacturer’s instruction (Thermo Fisher Scientific). The ligations mix was transformed into competent *E. coli* DH5α cells (Thermo Fisher Scientific) using a heat shock protocol [2] and then plated onto media containing 100 μg/mL spectinomycin. Plasmids were isolated from single *E. coli* colonies that were incubated overnight at 37°C using the GeneJET Plasmid Miniprep Kit (Thermo Fisher Scientific). The recombinant plasmids were then confirmed by PCR and Sanger sequencing at the Ramaciotti Centre for Genomics (University of New South Wales). The recombinant pSyn_6 plasmids were subsequently transformed into *S. elongatus* as per the guideline from the manufacturer (Thermo Fisher Scientific). Solid BG-11 agar plates supplemented with 1% agar and 100 µg/mL spectinomycin were used for colony selection. Following approximately 45 days of cultivation at 30°C, individual colonies were inoculated into liquid BG-11 media containing 100 µg/mL spectinomycin.

**Reverse-transcription polymerase chain reaction (RT-PCR)**

To confirm the expression of genes in the recombinant *S. elongatus*, total RNA was extracted using the modified TRIzol method with liquid nitrogen [3]. The ProtoScript® II First Strand cDNA Synthesis Kit (BioLab Inc., Watertown, MA, USA) was utilized for cDNA synthesis according to the manufacturer’s instructions. PCR was performed on both the purified DNAse-treated RNA and the cDNA using the primers pSyn_6-F2 and pSyn_6-R2. The absence of bands in the PCR with RNA confirmed the complete removal of DNA, while the presence of bands in the PCR with cDNA confirmed the expression of the target genes.

**Sponge interaction assay**

The sponge *Scopalina* sp. was collected from a depth of 9 meters at Bare Island, Botany Bay, New South Wales, Australia (33°59' S, 151°14' E). *Scopalina* sp. specimens were cut into cubes measuring 1.5 cm x 1.5 cm x 1 cm (roughly 2.25 g wet weight or 0.26 g of measured dry weight on average) and these explants were allowed to heal for at least a week in the aquarium at the Sydney Institute of Marine Science, Mosman, NSW, Australia. Healing was assessed before proceeding with the assay by examining the closure of the cut surfaces with a pinacoderm and the maintenance of the original colour.

*S. elongatus* was cultivated to the logarithmic growth phase and then acclimated for 24 hours in artificial seawater (ASW) that was autoclaved and filtered using a 0.22 μm membrane (Thermo Fisher Scientific). Healed sponge explants were transferred into a separate 500 mL incubation bottle containing 200 mL of ASW using sterile forceps under water to avoid air exposure. *S. elongatus* cells were added at a final concentration of 10^7^ cells/mL. This concentration was chosen for two reasons: (1) a lower concentration would make any decline during the experiment difficult to accurately quantify, and (2) and *Synechococcus* cells have been detected in concentration between 10^6^ and 10^7^ cells/mL in natural, marine blooms [4].

The incubation occurred under ambient laboratory lighting, 21°C and shaking at 100 rpm. Cyanobacterial concentrations in the ASW were quantified directly using a hemocytometer and dual-channel microscopy (brightfield and fluorescence) (Figure S6). Only single cells and no aggregates were observed. Each cyanobacterial clone was tested in triplicate, and the entire experiment was independently repeated twice. After incubation, explants were immediately processed for cryo-sectioning and confocal microscopy.

**Confocal microscopy**

For cryo-sectioning, sponge explants were directly transferred into 4% paraformaldehyde and incubated overnight at 4°C. After a brief rinse with 15% sucrose prepared in phosphate buffered saline (PBS), the specimens were further incubated in the same solution for 3 hours at 4°C. The buffer was then gradually replaced with Optimal Cutting Temperature (OCT) compound (Tissue Freezing Medium^®^, Leica Biosystems, Wetzlar, Germany) in a series of incubation with 15% sucrose/ OCT buffers at different ratios (35:10, overnight; 15:10 for 6h; 15:30 overnight and 100% OCT for 4 h) at 4°C. The specimens were then carefully transferred to disposable base moulds (Leica Biosystems) and frozen at -80°C overnight. Frozen samples were sectioned into 10 μm thick slices using a Epredia^™^ CryoStar^™^ NX70 Cryostat (Epredia/Thermo Fisher Scientific) and placed onto adhesion microscope slides (Menzel Gläser, SuperFrost® Plus, Thermo Scientific). Tissue slices were collected at varying depths through multiple rounds of sectioning. The prepared slides were stored at -80°C until further processing.

Prepared sponge slides stored at -80°C were allowed to equilibrate to room temperature. To ensure clear imaging surfaces without damaging the underlying sponge sections, excess frost or condensation was wiped off using a lint-free tissue. The slides were then stained with 1 ng/μL 4',6-diamidino-2-phenylindole (DAPI) for 15 minutes in the dark. The slides were then rinsed with ice-cold PBS to remove unbound dye and air-dried. Finally, a drop of standard antifade mountant (ProLong™ Diamond Antifade Mountant, Thermo Fisher Scientific) was applied, and a coverslip was carefully placed on top. Confocal microscopy was performed using an inverted LSM 900 microscope (Zeiss, Jena, Germany) equipped with a 63× oil-immersion objective lens (numerical aperture [NA] = 1.4) and a 0.5× digital zoom to optimize resolution and the field of view. Imaging was conducted in Airyscan mode, which enhances signal detection and resolution by reconstructing the image from 32-channel detector data, enabling superior detail in both lateral and axial dimensions. The optical section thickness is around 300 nm.

Fluorescence imaging of sponge cells and associated cyanobacteria was performed across three channels: excitation lasers at 405 nm (blue) for DAPI-stained sponge and microbial DNA, 488 nm (green) for cyanobacterial phycocyanin and sponge autofluorescence, and 561 nm (red) for cyanobacterial phycocyanin fluorescence. Each experimental condition included three replicates, with imaging conducted at two distinct depths per replicate. All three channels were merged and saved into a single file in CZI format to facilitate downstream analysis.

The blue and red channels were analyzed separately to quantify the number of sponge nuclei and cyanobacteria. First, the CZI files were imported into ImageJ [5] and all channels were split for independent analysis. Second, the brightness in each channel was adjusted using a colour threshold setting to eliminate background noise while retaining distinct DNA signals or cyanobacteria. Third, quantification was performed by filtering for particles with a size threshold above 2 μm with excitation lasers at 405 nm (sponge nuclei) and above 1 μm with excitation lasers at 561 nm (cyanobacteria). Nuclei and cyanobacteria signalos had minimal spatial overlaps and any visible clumps or anomalously large particles were manually excluded before quantification. Representative images were selected for localization analysis.

For Z-stack analysis, cryo-sectioning, staining, and imaging conditions followed the protocols described above. Z-stack images were acquired using a Zeiss LSM 980 microscope, with 15–20 optical sections per stack at a step size of ~0.8 µm. Z-stack analysis was performed on three cyrosections for every of three biological replicates. One random location per cryosection was analysed using the ZEN analysis software (Zeiss) with consistent threshold settings. Cyanobacteria were classified as intracellular when their red autofluorescence signal (see above) was entirely encapsulated by the sponge cell’s autofluorescence signal in the 488 nm-excited green channel across consecutive z-planes. Cyanobacteria were classified as extracellular when their signal was situated entirely outside the defined host boundaries (for examples, see Figure S5 and Supplementary Videos S1 and S2).

**Statistical analyses**

Statistical analyses were conducted using IBM SPSS Statistics version 22. As replicate-level variation was minor and no significant experiment × treatment interaction was detected, data from independent experiments were pooled within each treatment for the final analyses.

At each time point, bacterial concentrations (Figure 1b) were compared between treatments using a Poisson Generalized Linear Model (GLM) with a log link and treatment as a fixed factor. Model significance was evaluated by Wald χ² tests, and pairwise contrasts versus the negative control (*S. elongatus* with empty pSyn_6) were assessed at *p* < 0.05.

Differences in removal rates (Δcount/Δt) among treatments were analysed with a linear mixed-effects model, with treatment, time, and their interaction as fixed effects and sample ID as a random intercept to account for repeated measures. *A priori* contrasts comparing each treatment with the control at each time point were performed using Dunnett’s adjustment (via emmeans), and results are reported as estimated mean differences (treatment − control) with Dunnett-adjusted *p*-values. Model diagnostics showed no violations of normality or homoscedasticity. As sponge explants were cut to identical dimensions, rates were expressed per mL of incubation water without wet-weight normalization (which was not possible due to samples being directly processed for section).

Cyanobacterial counts derived from imaging (Figure 1c) were analyzed using a Poisson GLM with a log link and the log (sponge-nucleus count) as an offset to model the cyanobacteria-per-nucleus rate. Model assumptions for all GLMs were checked by inspecting Pearson residuals versus fitted values and testing for over-dispersion; residuals were randomly scattered without systematic deviation, indicating that the Poisson variance assumption was appropriate. All statistical tests were two-sided with a significance level of α = 0.05.

**Supplementary Results:**

For the *S. elongatus* clones with the recombinant pSyn_6 constructs, RT-PCR confirmed the expression of the ELP genes (Figure S1). PCRs with cDNA templates produced bands that correspond to the expected sizes of their respective genes, while no amplification was detected in the purified DNAse-treated RNA samples (Figure S1). Together, these findings show that the observed PCR amplifications originated from the cDNA and not from contaminating, residual DNA, confirming gene expression at the mRNA level.

Figure S1: RT-PCR analysis of ELP genes in recombinant *S. elongatus* strains. RNA from the strains containing the ELP gene was used as a control for the complete removal of DNA. The expected sizes of each ELP gene amplicon are shown in the brackets. From lane 1-11: Marker (lane 1), RNA for ELP9-expressing strain (lane 2), RNA for CAD42-expressing strain (lane 3), cDNA of ELP9-expressing strain (348 bp) (lane 4), cDNA of CAD42-expressing strain (547 bp) (lane 5), RNA for ELP1-expressing strain (lane 6), RNA for ELP5-expressing strain (lane 7), cDNA of ELP1-expressing strain (270 bp) (lane 8), ELP5-expressing strain (516 bp) (lane 9), RNA for SSA4-expressing strain (lane 10), cDNA of SSA4-expressing strain (2094 bp) (lane 11). Red arrows point to the specific bands for each ELP gene amplicon.

Figure S2: Fluorescence microscopy images of the tissue on *Scopalina* sp. based on blue 4',6-diamidino-2-phenylindole (DAPI) staining (upper image) and autofluorescence in red channel (lower image). Small bright-blue signals show microbial cells, which showed no defined red fluorescence above the autofluorescence of the sponge tissue. This indicates that the microbial cells are not cyanobacteria. Scale bar: 50 µm.

Figure S3: The concentration of *S. elongatus* in seawater expressing no recombinant protein in the presence (black) or absence (red) of *Scopalina* sp.. * indicates significant differences between treatments as determined using a Poisson generalized linear model (p < 0.05).

(b)

(c)

(d)

(a)

Figure S5: Example of a hemocytometer image to count *S. elongatus* cell in seawater used in the sponge interaction assay. Red autofluorescence of intact cyanobacteria is clearly visible and was used to determined cell concentrations.

**References**

1.	Nguyen MT, Liu M, Thomas T. Ankyrin‐repeat proteins from sponge symbionts modulate amoebal phagocytosis. *Molecular Ecology*. 2014;**23**:1635–45

2.	Froger A, Hall JE. Transformation of plasmid DNA into *E. coli* using the heat shock method. *Journal of Visualized Experiments*. 2007:253 <https://doi.org/10.3791/253>

3.	Kim Tiam S, Comte K, Dalle C *et al.* Development of a new extraction method based on high-intensity ultra-sonication to study rna regulation of the filamentous cyanobacteria *Planktothrix*. *PloS one*. 2019;**14**:e0222029

4.	Wang K, Wommack KE, Chen F. Abundance and distribution of *Synechococcus* spp. and cyanophages in the Chesapeake bay. *Applied and Environmental Microbiology*. 2011;**77**:7459–68 <https://doi.org/10.1128/AEM.00267-11>

5.	Schneider CA, Rasband WS, Eliceiri KW. Nih image to ImageJ: 25 years of image analysis. *Nature Methods*. 2012;**9**:671–75 <https://doi.org/10.1038/nmeth.2089>
